# COVID-19 Social Thermometer Project: transnational articulations to approach populations in situations of social vulnerability

**DOI:** 10.1590/0034-7167.202376suppl201

**Published:** 2023-02-06

**Authors:** Heriederson Sávio Dias Moura, Regina Célia Fiorati, Ricardo Alexandre Arcêncio

**Affiliations:** I Universidade de São Paulo, Escola de Enfermagem de Ribeirão Preto. Ribeirão Preto, São Paulo, Brazil.; II Universidade de São Paulo, Faculdade de Medicina de Ribeirão Preto. Ribeirão Preto, São Paulo, Brazil.

COVID-19 is an infectious disease that has significantly impacted different contexts around the world, directly and/or indirectly, and on a larger scale in poor and/or developing countries^(1)^. We do not know for sure the cumulative effects of COVID-19 on populations (mental disorders, insomnia, anxiety, thrombosis, cognition deficit, memory problems, etc.) after exposure to infection by the SARS-CoV-2 virus, mainly populations in a situation of social vulnerability (PSSV), which were the most affected, in addition to being those who do not know how they have managed to obtain resources and/or technologies to mitigate these effects and protect themselves against new episodes of infection and/or reinfection.

It is a fact that unhealthy conditions of high population density and substandard areas, such as slums, have been important niches for the high rate of transmissibility and infection by the virus^(2)^, mainly due to lack of access to the structure of social, economic and cultural opportunities that provide resources, putting certain groups at a disadvantage^(3, 4)^.

Thus, it became relevant to investigate how COVID-19 has impacted PSSV, given that, in Brazil, the number of people in extreme poverty has been increasing, especially after the adoption of austerity policies and cuts in Social Protection Programs.

To this end, a project was set up between the Portuguese National School of Health at the *Universidade Nova de Lisboa* (ENSP-UNL), the *Escola de Enfermagem de Ribeirão Preto* at the *Universidade of São Paulo* (EERP-USP) and the *Escola Nacional de Saúde Pública* at Fiocruz (ENSPFIOCRUZ). The first adaptation to the Brazilian context was to change the name of the project, which changed from Social Barometer (*Barómetro Social*) (Portugal) to Social Thermometer (*Termômetro Social*) (Brazil). In Brazil, the project took on transnational characteristics, with cultural adaptations and an appeal to ethical-political and humanitarian issues, given the announced catastrophe among vulnerable groups. To implement the project, a research cooperation network was created with the participation of researchers, workers, health managers and organized civil society (social movements and/or community organizations), from the five macro-regions of the country and foreigners (Portugal, United States, Spain, Chile, Argentina and Colombia).

Among the Social Thermometer's goals, the main one was to highlight the impacts of COVID-19 on the general population and PSSV residing in Brazil. We recognize the concern and importance of the project, especially for PSSV, given that they have fallen short of public policies, with very impaired access to health services, being at a greater disadvantage when compared to the general population. While we are all at risk of COVID-19 infection, the mechanisms and/or resources we have to protect ourselves from it and from the rehabilitation of sequel is different, which makes it a socially determined inequity.

The project has been advancing in all the capitals of the 26 states and the Federal District, using a hybrid methodology for applying an instrument to the reference population by a trained interviewer: online research with the general population, with a self-administered questionnaire, or offline, with field research in areas and populations without internet access, using for the latter the mobilization of organized civil society and/or other organizations, approaching four PSSV groups: homeless people, residents of slums and/or communities, of settlements and/or camps, and foreign migrants.

To date, we have had 2,293 respondents in the general population. Among PSSV groups, 751 people belong to the homeless population, 700 live in favelas and/or communities, 127 live in settlements and/or camps and 317 are foreign migrants. Some preliminary PSSV results show that: 1,138 (60%) of respondents were male; 1,324 (70%) were black or brown; about 562 (30%) people received some kind of government assistance; 1,140 (60%) individuals used TV news to stay informed about COVID-19; 779 (41%) consulted the internet; 482 (25%) consulted health professionals; 768 (40%) reported mental health status as well; 592 (31%) reported their mental health status as reasonable; 1,641 (87%) were vaccinated; and 81 (4%) of those who have not been vaccinated intend to be vaccinated.

The results have strong power to contribute to the formulation of public policies and generate protocols for health services, aiming at better care for these populations. In addition, project originality stands out, based on valuable aspects, such as the articulation with an interprofessional team and with organized civil society, which gives breadth to the investigation and allows the object of study to be analyzed from a social, cultural and political perspective. Approaching PSSV is extremely relevant in the project composition, as these populations need a holistic view, considering the individual and the collective subject, who lack objective and resolving strategies, considering the entire historical-structural context in which they are inserted.
